# The Impact of BiLevel Positive Airway Pressure (BiPAP) Application Timing on Emergency Room Length of Stay in Patients With Pulmonary Edema: A Single-Center, Retrospective Cohort Study

**DOI:** 10.7759/cureus.33193

**Published:** 2022-12-31

**Authors:** Sumaya Khayat, Majid Ali, Lama Almasoudi, Alaa Fatani, Walaa Albarnawi, Moayad Bsooki, Mohammad Ismail

**Affiliations:** 1 College of Medicine, Umm al Qura University, Makkah, SAU; 2 College of Medicine, Medical University of Warsaw, Warsaw, POL; 3 Respiratory Therapy Administration, King Abdullah Medical City, Makkah, SAU

**Keywords:** non-invasive ventilation, emergency medicine, pulmonary edema, chf, bipap

## Abstract

Background: Bilevel positive airway pressure (BiPAP) is a form of non-invasive ventilation (NIV) that is used to help and facilitate breathing. Our objective is to evaluate the impact of BiPAP application time on the length of emergency room (ER) stay in pulmonary edema patients.

Method: This is a retrospective cohort study that included patients who presented to the ER at King Abdullah Medical City (KAMC) from June 2019 to June 2021. The eligibility criteria for BiPAP application were congestive heart failure (CHF) and type 1 and type 2 respiratory failure, The data were collected from the Track Care system. We defined early BiPAP as BiPAP application time within one hour from admission, and late BiPAP more than one hour and we calculated the percentage of discharge within four hours in each group.

Result: Out of 147 fulfilling study eligibility, 64% had CHF, 23% had type 2 respiratory failure and 13% had type 1 respiratory failure. For patients discharged within four hours, 85% were in the early BiPAP and 15% were in the late BiPAP groups (p = 0.001 as compared to the late discharge group). Discharge within four hours was observed with the following percentages in the study subgroups: CHF early BiPAP (84%), late BiPAP (16%) (p = 0.004), type 1 respiratory failure early BiPAP (79%), late BiPAP (21%) (p = 0.71) and type 2 respiratory failure early BiPAP (94%), late BiPAP (6%) (p = 0.89).

Conclusion: Our results show that there is a significant outcome in early BiPAP application in decreasing the length of ER stay only in patients with pulmonary edema.

## Introduction

Bilevel-positive airway pressure (BiPAP) is a form of non-invasive ventilation (NIV) that is used to help and facilitate breathing [1,2]. It is a method that supports inspiration and helps decrease respiratory work. The pressure is higher during inspiration and lowers during expiration [3]. BiPAP uses to treat acute respiratory failure (ARF) and showed a significant reduction in mortality rate, decreasing endotracheal intubation, and length of stay compared with other type of therapies [4]. It is a less invasive alternative to endotracheal intubation for treating congestive heart failure (CHF). It has been found to improve oxygenation, and hemodynamic stability, and reduce the need for intubation in the treatment of CHF [5]. BiPAP has also been shown to improve respiration and vital signs in acute pulmonary edema patients faster than CPAP [6]. There are some advantages of BiPAP over endotracheal intubation such as speech, swallowing, avoiding trauma to the trachea and larynx, and patient comfort [7].

The length of hospital stays, in general, will increasingly bear the cost on health care providers [1]. According to a study done in China, early use of BiPAP less than 48 hours in patients with acute respiratory distress syndrome (ARDS) in the intensive care unit (ICU) improved oxygenation and respiratory system compliance while also shortening the time spent on mechanical ventilation and in the ICU.

Consequently, no studies have ever demonstrated that early BiPAP application could reduce hospital stay, and the current literature providing evidence for the use of NIV for preventing post-extubation respiratory failure and reintubation is limited [3]. In addition, Non-Positive Pressure Ventilation (NPPV) has been shown to be effective when used as an initial mode of assisted ventilation. A meta-analysis study suggests that NIV is worth considering as a treatment option for patients with ARF [4-7]. However, another meta-analysis study does not support the use of NPPV in hypoxemic ARF and ARDS and is advised to be cautiously used in patients with ARDS [5].

To my knowledge, there are a few studies focused on the impact of BiPAP application time on the length of stay in pulmonary edema patients [8]. There is insufficient information and inadequate experience concerning NIV and a limited number of subjects participating in the studies [9]. In addition, there are limited studies that mainly focus on CHF and respiratory failure 1 and 2 as indications for BiPAP use [10]. Studies that related to our research were old and needed newly updated data [11]. Accordingly, this research aimed to evaluate the impact of early application of BiPAP in decreasing hospital length of staying in pulmonary edema patients.

## Materials and methods

The study designed as a retrospective cohort study and was carried out between August and October of 2021 on patients with pulmonary edema who were admitted via the emergency department at King Abdullah Medical City (KAMC), a tertiary center, which is located in Makkah city.

The inclusion criteria included any patient receiving BiPAP for type 1 and 2 respiratory failure and patients with CHF. The exclusion criteria were any subject underage of 18, respiratory failure due to nonpulmonary pathology, impaired consciousness, or any patient with a contraindication to bilevel positive air pressure (BiPAP).

We used a convenience sampling technique with an estimated sample size to be 36.45. the data were collected from the Track Care system of the KAMC hospital database, the data collection process took place between June 2019 to June 2021. The study included 237 and excluded 163 of the participants with a total number of 400 patients.

Statistical analysis was performed using Statistical Package for the Social Sciences (SPSS) Version 26. A descriptive analysis of the data was performed. Numerical variables are presented as arithmetic median and interquartile range since they had abnormal distribution. Categorical data are presented as absolute numbers, and category frequency is expressed as percentages. The t-test was used to compare the groups. The level of significance was set at p < 0.05 or 5% to find a possible association between certain factors and the length of stay among the admitted patients.

The proposal of this study had been revised and approved by the ethics committee of King Abdullah Medical City (KAMC) with IRB number 21-812.

## Results

According to the collected data we found out that Most of the participants were males, 132 (55.7%). Saudi nationality represented most of the participants, 187 (78.9%) (Tables 1, 2).

**Table 1:** Percent of male and female involved Male is 55.7% and Female 44.3%.

		Frequency	Percent	Valid Percent	Cumulative Percent
Valid	Male	132	55.7	55.7	55.7
	Female	105	44.3	44.3	100.0
	Total	237	100.0	100.0	

**Table 2:** Percent of Saudi and non-Saudi involved Saudi is 78.9% and Non-Saudi 21.1%.

		Frequency	Percent	Valid Percent	Cumulative Percent
Valid	Saudi	187	78.9	78.9	78.9
	Non-Saudi	50	21.1	21.1	100.0
	Total	237	100.0	100.0	

**Figure 1:**
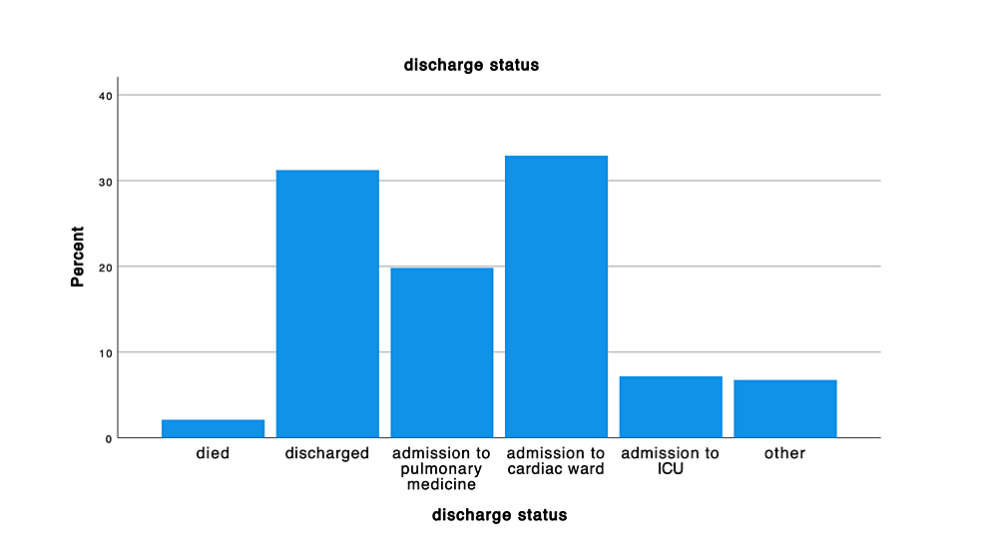
Discharge status

**Figure 2:**
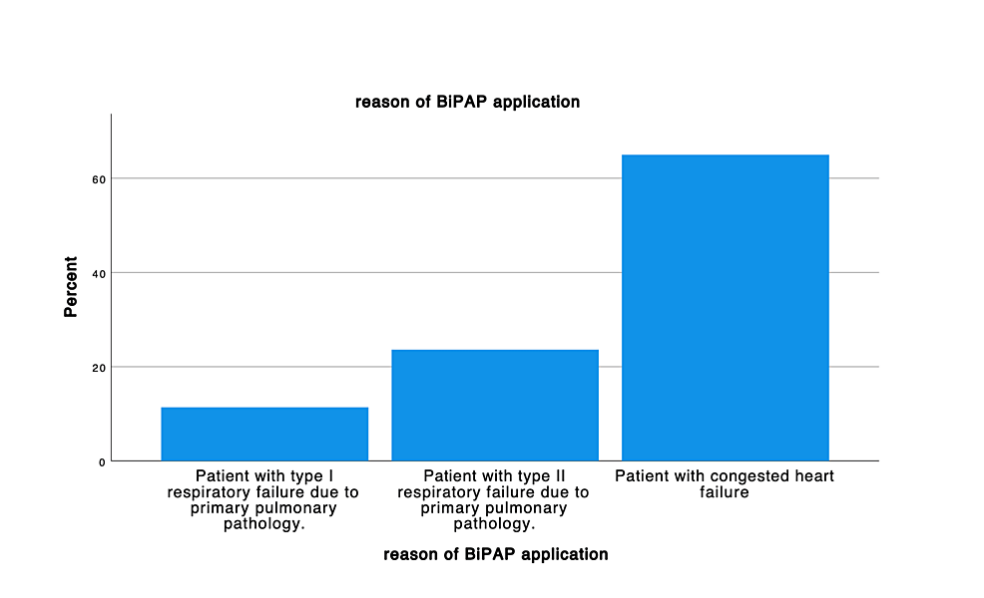
Reason of BiPAP application

The elderly population represented most of the participants, more than 61 years of age were 155(65.4%), and between 51 to 60 years of age were 53 (22.4%). In regard to the body mass index (BMI), the minority had normal to underweight BMI 50 (21.1%), and 2 (0.8%), respectively, were the majority represented an elevated BMI ranging from overweight 64 (27%), obese 71 (30%) and extreme obese 43 (18.1%). The discharge rate was highest in the cardiac unit, 78 (32.9%), followed by hospital discharge at 74 (31.2%) (Figure 1) and the primary reason for BiPAP application was CHF 154 (65%) (Figure 2).

Patients with CHF had an increased BMI in comparison to other indications of BiPAP administration, where 43 (27.9%), 51 (33.1%), and 27 (17.5%) patients represented overweight, obese, and extremely obese respectively. The elderly population was the most prevalent among all indications for BiPAP; 97 (63%) individuals with CHF, 39 (69.7%) having type 2 respiratory failure, and 19 (70.3%) with type 1 respiratory failure were above 61 years of age.

BiPAP application and target oxygen saturation: This study addresses 94% SpO_2_ as the target oxygen saturation. Out of 237 patients, more than half of them, 158 (66.3%) reached the target oxygen saturation (94%), while 79 (33.3) were not reached the target. Regarding BiPAP application and discharge time Out of 236 patients, 120 (50.8%) were on early BiPAP, whereas the remaining 116 (49.2%) used BiPAP after an hour. When comparing early versus late BiPAP application in terms of discharge time, we identified that 116 (56.6%) of those who had early BiPAP were discharged within four hours, while 89 (43.4%) were on BiPAP after one hour of admission.

Among 27 patients with type 1 respiratory failure, 10 (37%) patients were on early BiPAP, while the other 17 (63%) used BiPAP after an hour. Only 7 (33.3%) reached the target SpO_2_ within one hour after being administered BiPAP. On the other hand, 14 (66.6%) reach the target SpO_2_ after more than one hour. We observed that 10 (45.5%) of those who received early BiPAP were discharged before four hours, while 12 (54.5%) were removed after four hours.

Among Type 2 respiratory failure Out of 56 patients with type 2 respiratory failure, 37 (66.1%) were on early BiPAP, whereas the remaining 19 (33.9%) used BiPAP after an hour. More than half of the patients, 17 (68%), reached the target SpO_2_ within one hour after administering BiPAP, and 8 (32%) reached to target SpO_2_ after more than one hour.

In a comparison of early and late BiPAP application time, we identified that 37 (69.8%) of those who had early BiPAP were discharged within four hours, while 16 (30.2%) were discharged after four hours from admission. Out of 153 patients with congestive heart failure, 73 (47.7%) administered BiPAP in the first hour, while more than half of them after one hour, 80 (52.3%) (Table 3). Forty-nine (43.8%) of the patients reach the SpO_2_ target in one hour, and 112 (56.3%) reach the target SpO_2_ after one hour.

**Table 3:** Percentage of connecting BiPAP within one hour with discharge within four hours and connecting BiPAP after one hour with discharge after four hours

BiPAP in 1H * discharge within 4H Crosstabulation						
reason of BiPAP application				discharge within 4H		Total
				Yes	No	
Patient with congested heart failure	BiPAP in 1H	Yes	Count	69	4	73
			% within discharge within 4H	53.1%	17.4%	47.7%
		No	Count	61	19	80
			% within discharge within 4H	46.9%	82.6%	52.3%
	Total		Count	130	23	153
			% within discharge within 4H	100.0%	100.0%	100.0%

And according to discharge time, we address that 69 (53.1%) of the early BiPAP group were discharged within four hours, while 61 (46.9%) were discharged after four hours.

## Discussion

Most of the participants in the present study are from elder populations, with 65% being over 70 years old and, 89% being older than 51 years. This may reflect years of exposure to numerous risk factors, some of which could involve smoking, pollution, obesity, chronic diseases like hypertension or diabetes mellitus, or infections that may significantly impact heart and lung productivity, leading to more vulnerable status. In the present study, the majority had a high body mass index (BMI), thus provoking a greater need for ventilation modalities. A relevant article found that those with a high or morbid BMI had better outcomes compared to those with a normal BMI [12]. Of most participants, 92.50% were diagnosed with dyspnea related to CHF, with a lesser occurrence of both type 1 and 2 respiratory failures. Concerning the direct impact of BiPAP application, both heart rate and respiratory rate improved at the time of BiPAP application. Some reverse effects were documented on the oxygen partial saturation.

In CHF, early application of BiPAP reduced the length of emergency department stay (p < 0.05), which is consistent with a recent encounter in a study that reported NIV to be very effective in the reduction of intubation need along with overall mortality among CHF patients [13], and this might due the nature of the pathology as in heart failure the lung is intact but impacted by the congestion of the heart and the flow, whereas in respiratory failure the lung is primarily affected. A 2016 meta-analysis reported no significant association between noninvasive positive pressure ventilation (NPPV) among admitted patients or the length of an intensive care unit (ICU) or hospital stay. Gender and BMI did not impact the length of hospital stay, as many elderly patients may have similar disease courses over the years, but this is a recommendation and a gap for further research (Table 4) [14,15].

**Table 4:** Chi-square tests showing the p-value and the significant p-value It is shown the significance in connecting early BiPAP with early discharge (p < 0.05).

Reason for BiPAP applications	P-value
Patient with type I respiratory failure due to primary pulmonary pathology.	0.077
Patient with type II respiratory failure due to primary pulmonary pathology.	0.035
Patient with congested heart failure	0.001

As 116 (49.2%) of the total 236 patients were on early BiPAP, whereas the remaining 120 (50.8%) used BiPAP after an hour, this divided the sample into nearly two equal groups, which gives a more accurate assumption about the results. Generally, the BiPAP application time does not show significance regarding the discharge time. After classifying the patient according to the reason for BiPAP application, no significant found association between BiPAP application time and discharge time among types 1 and 2 respiratory failure patients. A meta-analysis performed on the NIV effect on acute respiratory failure supported our finding, which suggests that the usage of NPPV does not influence the length of hospital stay [16].

There was a significant change in the discharge time in patients with CHF, as 61 (46.9%) of the early BiPAP group were discharged within four hours, taking into consideration that cardiogenic pulmonary edema was the most common reason for BiPAP application in 153 (65%) of our sample. In contrast, other studies demonstrate either uncertainty about the effect of NPPV on the length of hospital stay [16] or that no difference was found [17] among CHF patients.

Among all participants, 158 out of 236 reached the target of SpO_2_ (94%), 85 patients (53.8%) received BiPAP within the first hour, and 73 (46.2%) applied BIPAP after the first hour passed. There was a slight improvement for those who received it within the first hour of the presentation.

There is a significant association of BiPAP in patients with type 1 respiratory failure compared to type 2, the highest indication for BiPAP was CHF which resulted in noticeable outcomes with BiPAP administration and SpO_2_ [18-20]. The largest body of evidence on the use of NIV comes from patients with chronic obstructive pulmonary disease (COPD) having exacerbation and respiratory failure [21-23]. NIV is effective in acute respiratory failure of various etiologies, therefore BiPAP effectively and safely provided early improvement and resolution of breath and SpO_2_ [18].

The strength of this study is the first study in Saudi Arabia. One of the limitations encountered was the lack of data and that it was conducted only in one tertiary hospital, which had only complicated cases. Furthermore, more than half of the participants were from the elderly population.

## Conclusions

Based on our findings, only patients with pulmonary edema due to CHF benefited from early BiPAP in shortening the duration of their hospital stay. The strength of this study is the first study in Saudi Arabia. Further research is encouraged to explore the efficacy of BiPAP administration in different settings and centers, along with possible associations between various etiologies or risk factors that may impact or stand as an obstacle to healthcare success and fruitful outcomes.
